# Comparison of correction formulas for intraocular pressure measured by Goldmann tonometer following various refractive surgeries (FS-LASIK, SMILE, tPRK)

**DOI:** 10.3389/fbioe.2026.1735079

**Published:** 2026-01-22

**Authors:** Jie Tong, Kai Zhou, HongJiang Wu, YeWei Zhao, ZhanHao Gu, YiQian Li, JiaHui Zong, XuanYa Tong, XiaoFei Zhou, LuMeng Wang, ShiHao Chen, Jia Qu, QinMei Wang, DongYe Xu, Ahmed Elsheikh, FangJun Bao

**Affiliations:** 1 National Clinical Research Center for Ocular Diseases, Eye Hospital, WenZhou Medical University, Wenzhou, China; 2 State Key Laboratory of Ophthalmology, Optometry and Vision Sicence, Eye Hospital, WenZhou Medical University, Wenzhou, China; 3 National Engineering Research Center of Ophthalmology and Optometry, Eye Hospital, WenZhou Medical University, Wenzhou, China; 4 ZheJiang-United Kingdom Joint Lab of Ocular Biomechanics, Wenzhou, China; 5 The Institute of Ocular Biomechanics, Wenzhou Medical University, Wenzhou, China; 6 OuJiang Laboratory, Eye Hospital, WenZhou Medical University, Wenzhou, China; 7 School of Engineering, University of Liverpool, Liverpool, United Kingdom; 8 National Institute for Health Research (NIHR) Biomedical Research Centre for Ophthalmology, Moorfields Eye Hospital NHS Foundation Trust and UCL Institute of Ophthalmology, London, United Kingdom; 9 Beijing Advanced Innovation Center for Biomedical Engineering, Beihang University, Beijing, China

**Keywords:** correction formula, dynamic contour tonometry, Goldmann applanation tonometer, intraocular pressure, refractive surgery

## Abstract

**Objective:**

This study aims to compare different intraocular pressure (IOP) correction formulas in post-refractive surgery patients who underwent Femtosecond Laser-Assisted *In Situ* Keratomileusis (FS-LASIK), Small Incision Lenticule Extraction (SMILE), and Trans-epithelial Photorefractive Keratectomy (tPRK), and to identify the most accurate formula for correcting IOP values measured by Goldmann applanation tonometer (GAT).

**Methods:**

This prospective study included 160 patients (160 eyes) who underwent FS-LASIK (58 eyes), SMILE (52 eyes), or tPRK (50 eyes) at the Eye Hospital, Wenzhou Medical University. IOP was measured using GAT and Dynamic Contour Tonometry (DCT) preoperatively and 3 months postoperatively. Optical zone diameter, ablation depth (AD), residual stromal thickness, refractive error correction, central corneal thickness (CCT), and mean corneal curvature (Km) were collected. Fourteen published GAT correction formulas (F1–F14) were applied to postoperative GAT values and compared with preoperative measurements. Differences between pre- and postoperative DCT readings served as a reference.

**Results:**

In FS-LASIK and SMILE, formulas F3, F4, and F11 showed superior correction performance. In FS-LASIK, the mean differences, concordance correlation coefficients (CCC), and proportions of differences within ±2 mmHg were closer to or better than the DCT reference (0.58, 0.572, 55.17%), with F3 (0.25, 0.372, 72.41%), F4 (0.20, 0.373, 63.79%), and F11 (0.04, 0.324, 67.24%) showing high agreement. Similarly, in SMILE, F3 (0.25, 0.379, 57.69%), F4 (0.22, 0.375, 61.54%), and F11 (0.01, 0.399, 63.46%) outperformed or approximated DCT (0.86, 0.447, 44.23%). In tPRK, F1 (0.18, 0.653, 64.00%) and F2 (0.25, 0.316, 62.00%) provided better correction than DCT (0.19, 0.493, 56.00%).

**Conclusion:**

Formulas F3, F4, and F11 are applicable for GAT IOP correction in both FS-LASIK and SMILE eyes. F1 and F2 are more suitable for tPRK; the preoperative IOP parameters included in F1 may lead to assessment bias, making F2 the recommended choice when preoperative IOP is unavailable.

## 1 Introduction

Intraocular pressure (IOP) represents the force exerted by the intraocular contents against the eye wall. Accurate assessment of IOP is crucial for the early diagnosis of glaucoma and for monitoring disease progression (De Francesco et al., 2024). In recent years, the prevalence of myopia has risen substantially, (Sun et al., 2023; Chong et al., 2023; Bull et al., 2023), and corneal refractive surgery has become a widely adopted method for correcting myopic refractive errors. Common procedures include Femtosecond Laser-Assisted *In Situ* Keratomileusis (FS-LASIK), Small Incision Lenticule Extraction (SMILE), and Trans-epithelial Photorefractive Keratectomy (tPRK). Although these procedures share the goal of achieving emmetropia, they differ in surgical mechanisms and all cause varying degrees of corneal biomechanical weakening (Xin et al., 2022; Hashemi et al., 2023). This biomechanical alteration can lead to an underestimation of IOP (Wang et al., 2019; Shin et al., 2015).

There are various types of tonometer used for measuring IOP, among which the Goldmann applanation tonometer (GAT) remains the clinical gold standard (Brusini et al., 2021). Corneal thickness has a significant influence on GAT readings, which are most accurate at 520 μm, and deviations can cause overestimation or underestimation of IOP (Schneider and Grehn, 2006; Andreanos et al., 2016). Dynamic Contour Tonometry (DCT) is less affected by corneal biometric properties such as thickness, tissue structure, and hydration. Studies have shown that changes in corneal thickness, curvature, and biomechanics after refractive surgery can cause a significant reduction in GAT-measured IOP values, while DCT is almost unaffected by postoperative corneal biomechanical changes (Aristeidou et al., 2011; Kaufmann et al., 2004). However, the clinical adoption and acceptance of DCT remain limited, partly due to its higher technical requirements and greater dependence on patient cooperation compared with GAT (Grieshaber et al., 2007; Schneider and Grehn, 2006).

Various GAT correction formulas have been proposed to adjust postoperative IOP readings. These formulas are mostly based on clinical data regression analysis, (Chihara et al., 2005; Duch et al., 2001; Helmy and Hashem, 2020; Rashad and Bahnassy, 2001),mathematical analysis, or finite element simulations (Asejczyk-Widlicka and Srodka, 2020). Some were derived from patients who have undergone Laser *In Situ* Keratomileusis (LASIK), (Chihara et al., 2005; Duch et al., 2001; Helmy and Hashem, 2020; Rashad and Bahnassy, 2001; Emara et al., 1998; Svedberg et al., 2005; Arimoto et al., 2002), while others were developed using data from patients who underwent Photorefractive Keratectomy (PRK) (Svedberg et al., 2005; Arimoto et al., 2002; Munger et al., 1998; Rosa et al., 1998). De Bernardo et al. conducted a comparative evaluation of nine correction formulas, (De Bernardo et al., 2016; Yang et al., 2022), assessing their effectiveness for postoperative IOP adjustment after PRK and identifying the one that demonstrated the best performance. However, their optimal correction approach relied on averaging two formulas, and their analysis was limited exclusively to PRK cases. There is currently no universally recognized method for comparing the effectiveness of postoperative IOP correction formulas in refractive surgery. Moreover, comprehensive comparisons of the applicability of GAT-IOP correction formulas for FS-LASIK, SMILE, and tPRK surgeries remain lacking.

In this study, we compared GAT-measured IOP values at 3 months after surgery corrected by different formulas with the preoperative GAT-measured IOP values. Differences in DCT readings, which are minimally influenced by refractive surgery, were also analyzed before and after surgery and used as reference standards. By employing multiple comparative metrics, we aimed to determine the optimal IOP correction formula for myopic patients undergoing FS-LASIK, SMILE, or tPRK. The findings of this study may contribute to more precise postoperative IOP assessment and help reduce the risk of misdiagnosing ocular hypertension and glaucoma due to inaccurate IOP measurements following corneal refractive surgery.

## 2 Methods

### 2.1 Patients and parameters

This prospective study included 160 myopic patients (160 eyes) who were scheduled to undergo one of three refractive surgeries—FS-LASIK (58 eyes), SMILE (52 eyes), or tPRK (50 eyes)—at the Eye Hospital, Wenzhou Medical University. The inclusion criteria were that all patients were of East Asian and Han ethnicity, diagnosed with refractive errors, and met the surgical indications for corneal refractive surgery. Patients were excluded if they had other ocular diseases, a history of ocular surgery or trauma, systemic disorders, discontinued contact lens wear less than 2 weeks before surgery, were unwilling to participate, or were unable to complete the 3-month postoperative follow-up. This study followed the principles of the Helsinki Declaration and was approved by the Institutional Review Board of the Eye Hospital, Wenzhou Medical University (KYK [2018]29).

Surgical parameters were recorded from surgical plans and treatment outputs, including optical zone diameter, ablation depth (AD), residual stromal thickness, and the degree of refractive correction expressed as the corrected mean spherical equivalent (cMSE). Central corneal thickness (CCT) and the mean curvature power in the central 3 mm of the anterior surface (Km) were measured with corneal topography (Pentacam HR; Oculus Optikgeräte GmbH). GAT (AT900, Haag-Streit) and DCT (SMT Swiss Microtechnology AG) IOP data were measured before and 3 months after surgery, recorded as IOPpre and IOPpost. Examinations were performed by an experienced examiner during the same half-day session, with patients in a seated position. Both DCT and GAT use contact method. The measurement sequence was randomized and contact tonometry was performed under surface anesthesia. Each measurement was repeated three times with at least 5 minutes between measurements, and the IOP value was taken as the average of readings differing by less than 2 mmHg.

Fourteen different Goldmann tonometer correction formulas (F1 to F14) were collected from different literature and are summarized in Table 1. Three-month postoperative GAT-measured IOP values were adjusted using these 14 formulas and compared with preoperative GAT measurements. DCT measurements before and after surgery were also compared as reference.

**TABLE 1:** Fourteen Goldmann tonometer correction formulas.

Number	Original formula
F1 (16)	$IOPc=IOPpost-6.455+\left(0.596*IOPpre\right)$
F2 (24)	IOPc=IOPpost+2.1*ΔCCT/100
F3 (21)	IOPc=IOPpost+ΔCCT/37.8
F4 (17)	IOPc=IOPpost+1.59+0.019*ΔCCT
F5 (25)	$IOPc=IOPpost+\left(0.025*AD\right)+\left(0.34*D\right)$
F6 (22)	IOPc=IOPpost+4.340+0.018*ΔCCT−0.440*ΔK
F7 (46)	$IOPc=IOPpost+\left(540-CCTpost\right)/71+\left(43-Kpost\right)/2.7+0.75$
F8 (20)	$\begin{array}{l} IOPc=\left[\left(0.55-Rpost/7.8*CCTpost\right)+1\right]*\\ \left(-1.61+0.94*IOPpost+0.0111*IOPpost*IOPpost+3.4\right)-3.4 \end{array}$
F9 (18)	IOPc=4+0.7*IOPpre−0.03*AD
F10 (Kohlhaas et al., 2006b)	$IOPc=IOPpost+\left(-0.0423*CCTpost\right)+23.28$
F11 (23)	IOPc=IOPpost+0.7−0.4*D
F12 (23)	IOPc=IOPpost+1.8−0.3*D
F13 (19)	IOPc=0.987+0.627*IOPpre+0.0143*ΔCCT+0.03044*age
F14 (22)	IOPc=IOPpost+2.765−0.001*ΔCCT−0.424*ΔK

IOPc: The IOP, corrected by the correction formula 3 months after the operation; IOPpre: the IOP, values measured preoperatively with the GAT; ΔCCT: preoperative - postoperative central corneal thickness values; Rpost: postoperative central anterior radius; AD: ablation depth; D: refractive error correction degree converted to corrected mean spherical equivalent; ΔK: preoperative–postoperative the mean curvature power in the central 3 mm of the anterior surface. F1 to F14 means corrected formula 1 to formula 14 to correct Goldmann tonometer IOP, measurement after corneal refractive surgeries.

### 2.2 Surgery

In FS-LASIK surgery, flap creation was performed with a femtosecond laser (Ziemer Ophthalmic Systems AG) and tissue ablation was performed using the Amaris 750 Hz excimer laser (Schwind eye-tech-solutions). The flap thickness ranged from 95 to 110 μm, and the ablation zone diameter from 6.93 mm to 8.35 mm. SMILE procedures were performed using a femtosecond laser platform (VisuMax; Carl Zeiss Meditec, Jena, Germany), in which a stromal lenticule with a thickness of 68–145 μm was extracted beneath a 120 μm cap. In tPRK surgery, epithelial and stromal ablation was performed in an aberration-free mode of the Schwind Amaris 750 Hz excimer laser, with cutting depth of 49–132 μm.

Postoperative care for all three surgeries was similar: postoperatively, a drop of tobramycin-dexamethasone was administered, and a bandage contact lens was placed on the cornea, to be kept for 1 day after FS-LASIK surgery, and 5–7 days after tPRK surgery until complete corneal re-epithelization. Following bandage lens removal, 0.1% fluorometholone and 0.5% levofloxacin were used four times daily for 1 week. Subsequently, the dose of fluorometholone was gradually reduced weekly and discontinued after 1 month in FS-LASIK and SMILE surgeries. In the tPRK group, the dose of fluorometholone was gradually reduced over 2–3 weeks postoperatively and discontinued after 2–3 months.

### 2.3 Statistical analysis

Statistical analysis was performed using SPSS 26.0 and MedCalc. One-way ANOVA or the Kruskal–Wallis test was used to compare the differences among the three refractive surgeries according to a normal distribution test. Differences between preoperative and postoperative IOP measurements were evaluated using paired t-tests and Cohen’s-D, an effect size statistic that standardizes mean difference (smaller Cohen’s-D values indicate smaller difference). The concordance correlation coefficient (CCC), which combines properties of the Pearson correlation coefficient and the impact of bias, was used to assess both correlation and accuracy. Bland-Altman plots were used to assess the level of agreement between preoperative and postoperative IOP. The percentage of prediction errors within ±2 mmHg was used as a comprehensive indicator to compare the performance of different correction formulas.

## 3 Results

The preoperative data of the included patients are presented in Table 2. There were no significant differences in age, cMSE, preoperative CCT, preoperative GAT and DCT intraocular pressure values among the three surgical methods. However, there were statistically significant differences (P < 0.05) in ablation depth (AD) and preoperative Km. Table 3 shows the postoperative GAT and DCT readings, as well as postoperative GAT values corrected using various formulas. Overall, uncorrected GAT yielded lower postoperative IOP values, whereas DCT produced higher measurements. Most correction formulas produced postoperative IOP values that lay between those obtained with uncorrected GAT and DCT; however, F8 and F9 showed postoperative IOP values slightly lower than those measured by uncorrected GAT across all three procedures.

**TABLE 2:** Baseline characteristics of the eyes by group.

Parameter	FS-LASIK (n = 58)	SMILE (n = 52)	tPRK (n = 50)	P value
Age (year)	25.95 ± 6.14	27.54 ± 5.70	26.84 ± 4.23	0.430
cMSE (D)	−5.90 ± 1.73	−5.41 ± 1.63	−5.91 ± 2.05	0.273
AD (μm)	100.22 ± 39.65	110.65 ± 20.32	88.40 ± 22.29	<0.05
CCT_pre_ (μm)	545.77 ± 17.61	547.41 ± 15.80	541.89 ± 24.79	0.059
K_mpre_ (D)	43.24 ± 1.39	43.49 ± 1.46	43.90 ± 1.20	0.043
GAT-IOP_pre_ (mmHg)	13.40 ± 1.95	13.17 ± 1.92	12.69 ± 2.33	0.197
DCT-IOP_pre_ (mmHg)	17.27 ± 2.47	17.55 ± 2.42	16.80 ± 2.85	0.341

cMSE, corrected mean spherical equivalent; AD, ablation depth; pre means at pre surgery stage; CCT, central corneal thickness; Km: mean curvature power in the central 3 mm of the anterior surface; GAT: intraocular pressure measured by goldmann applanation tonometer; DCT: Intraocular pressure measured by Dynamic Contour Tonometry. pre means at pre surgery stage.

Numbers in parentheses indicate the corresponding reference numbers for each correction formula.

**TABLE 3:** Three-month postoperative GAT and DCT measurements, and formula-corrected postoperative IOP: mean (±standard deviation).

Different IOP value, mmHg	FS-LASIK	SMILE	tPRK
GAT-IOP_pos_	10.41 ± 2.08	10.28 ± 1.96	11.08 ± 2.42
DCT-IOP_pos_	15.90 ± 2.25	15.55 ± 2.25	16.29 ± 2.57
IOP_c-F1_	11.94 ± 2.72	11.68 ± 2.60	12.18 ± 3.17
IOP_c-F2_	12.39 ± 2.04	12.19 ± 1.99	13.27 ± 2.31
IOP_c-F3_	12.90 ± 2.05	12.68 ± 2.01	13.84 ± 2.31
IOP_c-F4_	13.79 ± 2.04	13.60 ± 1.98	14.65 ± 2.32
IOP_c-F5_	10.91 ± 2.38	11.21 ± 1.98	11.28 ± 2.47
IOP_c-F6_	14.30 ± 2.04	14.39 ± 1.92	15.09 ± 2.53
IOP_c-F7_	14.14 ± 2.04	13.59 ± 2.06	14.79 ± 2.37
IOP_c-F8_	9.94 ± 2.57	9.86 ± 2.49	11.06 ± 2.99
IOP_c-F9_	10.37 ± 1.80	9.90 ± 1.31	10.23 ± 1.86
IOP_c-F10_	14.58 ± 2.17	14.24 ± 2.25	15.85 ± 2.53
IOP_c-F11_	13.47 ± 2.20	13.15 ± 2.10	14.14 ± 2.33
IOP_c-F12_	13.98 ± 2.15	13.71 ± 2.05	14.65 ± 2.33
IOP_c-F13_	11.52 ± 1.27	11.38 ± 1.27	11.25 ± 1.46
IOP_c-F14_	11.02 ± 2.19	11.16 ± 1.95	11.61 ± 2.75

pre means at pre surgery stage; post means at post surgery stage; IOPc: postoperative GAT-measured IOP, corrected by formula.; GAT: intraocular pressure measured by goldmann applanation tonometer; DCT: intraocular pressure measured by dynamic contour tonometry.

As shown in Table 4, postoperative IOP values corrected by F3, F4, F11, and F12 showed no statistically significant differences compared to preoperative IOP values in the FS-LASIK and SMILE groups (all p > 0.05, with small Cohen’s-D). In the tPRK group, postoperative IOP values corrected by F1 and F2 did not differ significantly from preoperative IOP values (all p > 0.05). Meanwhile, DCT measurements differed significantly before and after surgery in the FS-LASIK and SMILE groups (all p < 0.05), but not in the tPRK group (p > 0.05).

**TABLE 4:** Comparison between preoperative and postoperative IOP measurements calculated using different formulas in different surgical groups.

Measurement (Formula)	FS-LASIK		SMILE		tPRK	
​	P value	D value	P value	D value	P value	D value
GAT-IOP_dif_	<0.05	1.48	<0.05	1.49	<0.05	0.68
DCT-IOP_dif_	<0.05	0.58	<0.05	0.86	0.183	0.19
GAT-IOP_dif-F1_	<0.05	0.62	<0.05	0.65	0.125	0.18
GAT-IOP_dif-F2_	<0.05	0.51	<0.05	0.51	0.133	0.25
GAT-IOP_dif-F3_	0.094	0.25	0.108	0.25	<0.05	0.50
GAT-IOP_dif-F4_	0.182	0.20	0.166	0.22	<0.05	0.84
GAT-IOP_dif-F5_	<0.05	1.14	<0.05	1.01	<0.05	0.59
GAT-IOP_dif-F6_	<0.05	0.45	<0.05	0.63	<0.05	0.99
GAT-IOP_dif-F7_	<0.05	0.37	0.194	0.21	<0.05	0.90
GAT-IOP_dif-F8_	<0.05	1.51	<0.05	1.49	<0.05	0.61
GAT-IOP_dif-F9_	<0.05	1.62	<0.05	1.99	<0.05	1.17
GAT-IOP_dif-F10_	<0.05	0.58	<0.05	0.51	<0.05	1.30
GAT-IOP_dif-F11_	0.810	0.04	0.935	0.01	<0.05	0.62
GAT-IOP_dif-F12_	0.630	0.29	0.083	0.27	<0.05	0.84
GAT-IOP_dif-F13_	<0.05	1.14	<0.05	1.10	<0.05	0.74
GAT-IOP_dif-F14_	<0.05	1.15	<0.05	0.65	<0.05	0.42

D value: Cohen’s-D value, the size of the mean difference between two groups. IOP, _dif-F1_ to IOP, _dif-F14_: differences between formula-corrected postoperative GAT-IOP, and preoperative IOP.

**FIGURE 1:**
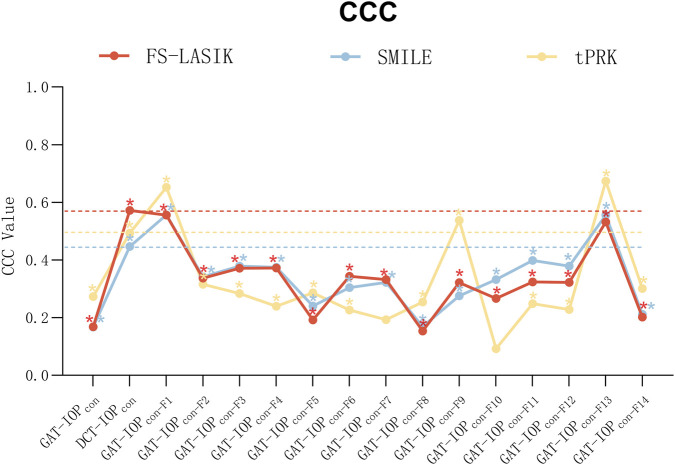
Comparison of concordance correlation coefficient(s) (CCC) of three surgical methods (GAT-IOP_con_ = Consistency of IOP measured by Goldmann tonometer before and after operation; DCT-IOP_con_ = Consistency of IOP measured by Dynamic Contour Tonometry before and after operation; IOP_con_-F1 to IOP_con_-F14 = The consistency of preoperative IOP measurements by Goldmann applanation tonometer and postoperative IOP corrected using Formula 1 to Formula 14. The dashed line serves as a reference for comparing the IOP measured by Dynamic Contour Tonometry postoperatively and preoperatively.).

The CCC is illustrated in Figure 1. F1 and F13 demonstrated consistently superior agreement across FS-LASIK, SMILE, and tPRK, with CCC values comparable to or exceeding those of DCT. Formula ranking patterns were similar between FS-LASIK and SMILE but differed from those observed in tPRK. F3 and F4 demonstrated good performance in FS-LASIK (CCC = 0.372 and 0.373, respectively) and SMILE (CCC = 0.379 and 0.375, respectively); however, their CCC values were lower than those observed with DCT. In contrast, tPRK demonstrates different results, with F9 (CCC = 0.538) and F2 (CCC = 0.316) showing best consistency coefficients except for F1 and F13. Across all three procedures, most correction formulas achieved higher CCC values than uncorrected GAT measurements, except for F8 in FS-LASIK (CCC = 0.153) and F10 in tPRK (CCC = 0.0923). The CCC improvements for all other formulas were statistically significant (all P < 0.05), whereas F7 and F10 in tPRK did not reach significance.

**FIGURE 2:**
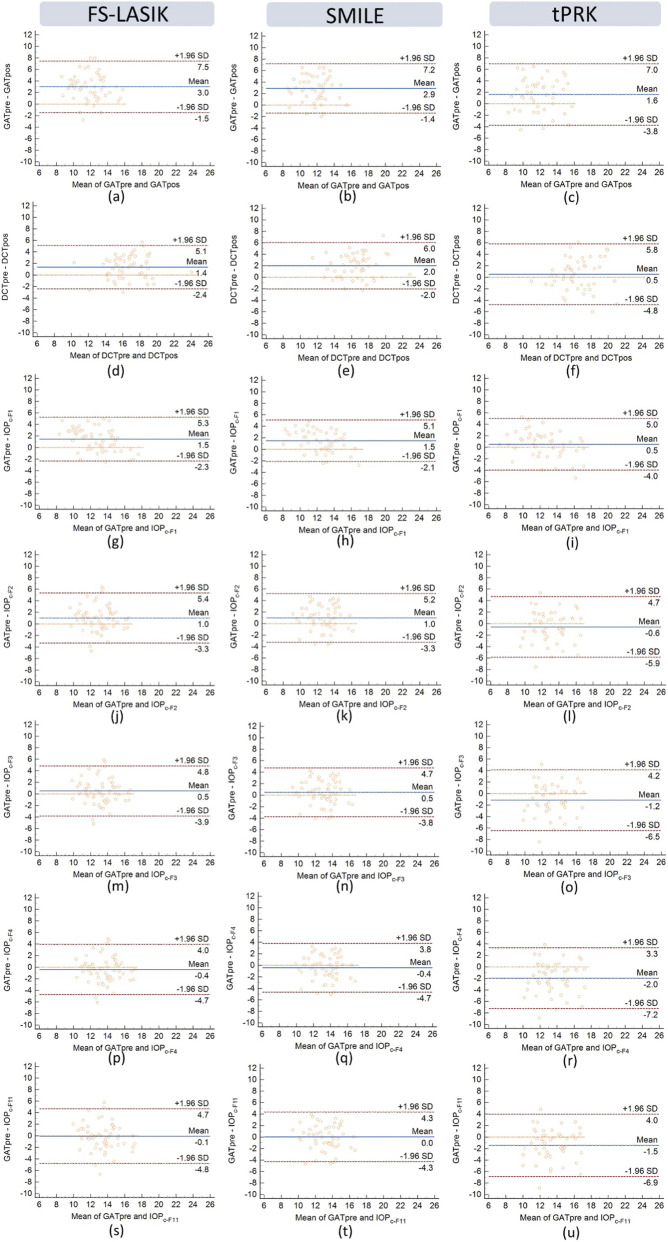
Bland Altmann analyzed the IOP measurement results of GAT **(a–c)** and DCT **(d–f)** before and 3 months after the three types of surgery (FS-LASIK, SMILE and tPRK), as well as the GAT-pre and the GAT-post corrected using formula 1 **(g–i)**, formula 2 **(j–l)**, formula 3 **(m–o)**, formula 4 **(p–r)**, and formula 11 **(s–u)**.

Due to the large number of Bland-Altman plots for all formulas across the three surgical methods (FS-LASIK, SMILE, tPRK), Figure 2 presents only those with relatively better consistency. These plots illustrate the mean difference (Δ = preoperative - postoperative IOP values) and the 95% limits of agreement (LoA) between postoperative IOP values corrected by each formula and preoperative values. The average differences in pre and post-operative IOP measured by DCT for all three surgical procedures are closer to zero compared to GAT, although both show similarly narrow LoA. After correction by F1, the average differences are closer to zero in both SMILE and tPRK than with DCT, with narrower LoA, and similar to DCT in FS-LAISK. After correction using F3, F4, and F11, the mean pre–post differences in FS-LASIK and SMILE eyes tended to be closer to zero than those obtained with uncorrected GAT and were also smaller than the corresponding DCT differences. Although the limits of agreement after correction were slightly wider than those of DCT, they remained narrower than those of uncorrected GAT for F3 and F4. In the tPRK group, postoperative values corrected by F1 and F2 show consistency comparable to DCT. Except for the data shown in Figure 2, the 95% LoA after correction with F8 is quite large for all three surgical methods (FS-LASIK: from −1.72–8.62 mmHg; SMILE: from −1.61–8.23 mmHg; tPRK: from −4.59–7.84 mmHg), and the mean differences are also large (FS-LASIK: 3.45 mmHg; SMILE: 3.31 mmHg; tPRK: 1.62 mmHg). F9 also has a narrower 95% LoA across the three surgical methods (FS-LASIK: from 0.41 to 5.63 mmHg; SMILE: from 1.42 to 5.12 mmHg; tPRK: from 0.72 to 4.20 mmHg), and the mean differences are larger (FS-LASIK: 3.02 mmHg; SMILE: 3.27 mmHg; tPRK: 2.46 mmHg).

**FIGURE 3:**
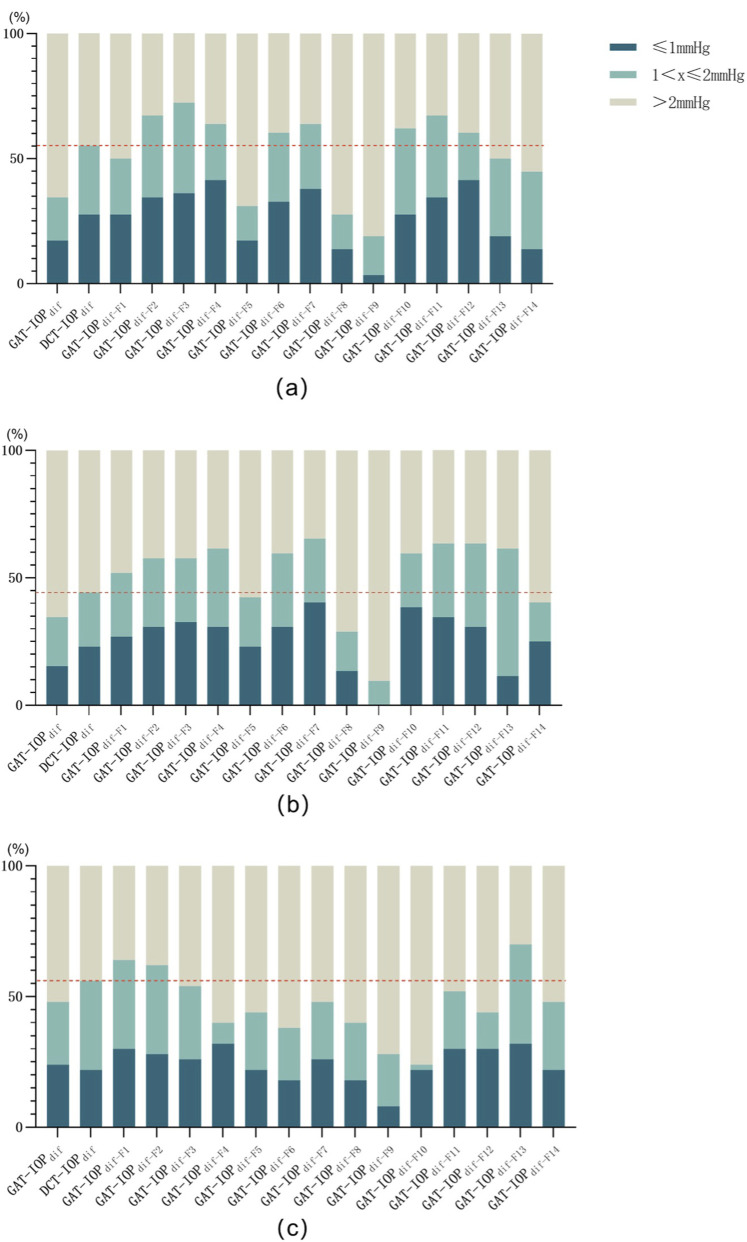
The percentage of predicted errors within 2 mmHg after FS-LASIK **(a)**, SMILE **(b)**, and tPRK **(c)** using different correction formulas. (GAT-IOP_dif_ = The difference between preoperative and postoperative IOP measured by Goldmann tonometer; DCT-IOP_dif_ = The difference between preoperative and postoperative IOP measured by Dynamic Contour Tonometry; IOP dif-F1 to IOPdif-F14 means the difference between the IOP measured by Goldmann tonometer after corneal refraction using formulas 1 to 14 and the preoperative IOP measured. The dashed line serves as a reference for comparing the IOP measured by Dynamic Contour Tonometry postoperatively and preoperatively.)

Figure 3 shows the difference between preoperative and formula-corrected postoperative IOP, and the proportion of the difference within the range of ±2 mmHg. In the FS-LASIK group, the highest proportions of differences within ±2 mmHg were observed for F3 (72.41%), followed by F11 (67.24%), F2 (67.24%), and F4 (63.79%). In the SMILE group, F7 (65.38%) showed the highest proportion of differences within ±2 mmHg, followed by F11 (63.46%), F12 (63.46%), F4 (61.54%), and F13 (61.54%). For tPRK, many formulas result in increased errors compared to DCT. F13 (70.00%), F1 (64.00%) and F2 (62.00%) exhibit relatively better performance, with errors within 2 mmHg.

## 4 Discussion

Corneal refractive surgery, which removes part of the cornea to focus light on the retina, alters corneal biomechanics and leads to postoperative IOP underestimation by the Goldmann tonometer (Chatterjee et al., 1997). Although many investigators have proposed correction formulas, most studies focus on PRK, and there is still no comprehensive formula collection or thorough comparative analysis for FS-LASIK, SMILE, and tPRK surgeries. The evaluation indicators for these formulars are also limited. In our study, we complied 14 previously published GAT correction formulas for these three surgical methods and introduced new evaluation indicators—including the CCC and the proportion of the difference within 2 mmHg—to identify the most suitable formula for each. What’s more, we used the DCT-IOP difference as a relative methodological reference. If the difference after formula correction is smaller than the DCT difference, we consider the correction effect to be excellent.

GAT and DCT are both contact methods for measuring intraocular pressure, and their measurement results are highly correlated (Ceruti et al., 2009). Studies by Siganos et al. (2004) and Aristeidou et al. (2011) showed that DCT measurements remain unchanged by refractive surgery, indicating no significant difference between preoperative and postoperative values across multiple surgical procedures (myopic PRK, myopic LASIK and hyperopic LASIK). Although DCT measurements may still be influenced by factors such as age and corneal curvature, (Xin et al., 2022; Yalcinbayir et al., 2010), accumulating evidence suggests that DCT is substantially less affected by corneal thickness (Schneider and Grehn, 2006; Ku et al., 2006) and biomechanical alterations (Aristeidou et al., 2011; Kaufmann et al., 2004; Kaufmann et al., 2003) and shows closer agreement with invasive manometric measurements (Kanngiesser et al., 2005; Kniestedt et al., 2005; Boehm et al., 2008; Kniestedt et al., 2004) than GAT. Consequently, DCT has been widely adopted as a relative reference standard in noninvasive comparative studies (Wachtl et al., 2017). Therefore, we used the DCT-IOP difference as one of the reference standards. If the difference after formula correction is close to or even smaller than the DCT difference, we consider the correction effect to be good. In our study, the difference in DCT before and after surgery was smaller in tPRK (p = 0.183, Cohen’s d value = 0.19) compared to FS-LASIK (p < 0.05, Cohen’s d value = 0.58) and SMILE (p < 0.05, Cohen’s d value = 0.86). This may be due to the fact that tPRK surgery has the least biomechanical impact on the cornea. In the FS-LASIK and SMILE groups, pre–post differences measured by DCT were smaller than those observed with GAT, indicating that DCT is less affected by refractive surgery–related corneal alterations. However, DCT measurements may still be influenced by corneal status, physiological IOP fluctuations, and inherent measurement variability. Accordingly, in the present study, DCT was not regarded as a gold standard but was used as a reference for change, allowing for a contextual assessment of the correction formulas.

The CCC primarily reflects the consistency between two variables, including their correlation and the impact of bias, providing a single, quantified value (Lin, 1989). Bland–Altman analysis evaluates agreement from a clinical perspective by explicitly visualizing systematic bias and the limits of agreement (LoA), thereby indicating the expected range of error for individual measurements (Giavarina, 2015). Although related, these two approaches assess complementary dimensions of agreement, and discrepancies between them may occur when measurements exhibit minimal bias but substantial variability, or *vice versa* (Kottner et al., 2011). Therefore, CCC should be interpreted in conjunction with Bland–Altman bias and LoA, as clinical applicability is more appropriately determined by absolute agreement and clinically acceptable error margins rather than by a single concordance metric alone. Schipper et al. has found that the IOP dropped by 2–3 mmHg after PRK, (Schipper et al., 1995), so we calculated the proportion of the difference within 2 mmHg for each formula to provide more precision support. Based on a comprehensive evaluation incorporating mean difference, dispersion, effect size, CCC, and the proportion within ±2 mmHg, several correction formulas demonstrated performance comparable to or exceeding that of DCT within specific surgical subgroups. In FS-LASIK, the difference between preoperative and postoperative DCT measurements was compared (1.36 ± 1.92 mmHg, 0.58, 0.572, 55.17%) (mean ± SD, Cohen’s d value, CCC value, the proportion of the difference within 2 mmHg). The differences between preoperative GAT and postoperative measurements using F3 (0.50 ± 2.22 mmHg, 0.25, 0.372, 72.41%), F4 (−0.39 ± 2.22 mmHg, 0.20, 0.373, 63.79%), F6 (−0.90 ± 2.22 mmHg, 0.45, 0.344, 60.34%), F7 (−0.73 ± 2.26 mmHg, 0.37, 0.332, 63.79%), F11 (−0.08 ± 2.41 mmHg, 0.04, 0.324, 67.24%), and F12 (−0.59 ± 2.36 mmHg, 0.29, 0.322, 60.34%) were closer to or even smaller than those of DCT. In SMILE, the differences between preoperative GAT and postoperative measurements using, F3 (0.49 ± 2.16 mmHg, 0.25, 0.379, 57.69%), F4 (−0.42 ± 2.16 mmHg, 0.22, 0.375, 61.54%), F7 (−0.42 ± 2.30 mmHg, 0.21, 0.322, 65.38%), F11 (0.03 ± 2.20 mmHg, 0.01, 0.399, 63.46%), and F12 (−0.53 ± 2.18 mmHg, 0.27 0.379, 63.46%) were closer or even better than the DCT difference (2.00 ± 2.06 mmHg, 0.86, 0.447, 44.23%). In tPRK, the differences between preoperative GAT and postoperative measurements from F1 (0.50 ± 2.28 mmHg, 0.18, 0.653, 64.00%) and F2 (−0.58 ± 2.70 mmHg, 0.25, 0.316, 62.00%) was closer or even better than the DCT difference (0.52 ± 2.71 mmHg, 0.19, 0.493, 56.00%). In the LASIK-based models (F1, F3, F4, F6, F7, F9, F12, F13), F3, F4, F7, and F12 showed better corrective outcomes after FS-LASIK and SMILE but only average performance for tPRK. F6 produced moderate outcomes, and F9 performed poorly. In contrast, F1 did not show a significant advantage in FS-LASIK and SMILE but performed better in tPRK. In the PRK-based (F2, F5, F11, F14), F2 performed better in tPRK, while F11 performed better in FS-LASIK and SMILE.

In our study, there was partial overlap in the correction formulas that performed well after FS-LASIK and SMILE, whereas more pronounced differences were observed when compared with tPRK. This discrepancy is likely attributable to both differences in the methodological frameworks used to derive the correction models and the distinct biomechanical alterations induced by each refractive procedure. The correction formulas were originally developed based on different surgical techniques, study populations, and modeling assumptions, which may limit their generalizability across procedures. From a biomechanical perspective, FS-LASIK and SMILE induce more substantial modifications to corneal structure than tPRK, including greater disruption of stromal collagen architecture, changes in residual stromal thickness, and redistribution of corneal stress (Bao et al., 2023; Sinha Roy and Dupps, 2011). In contrast, tPRK avoids flap or lenticule creation and primarily affects the superficial stroma, thereby preserving more of the anterior corneal architecture and resulting in a smaller magnitude of biomechanical alteration (Xin et al., 2022; Pniakowska et al., 2022). These surgery-specific biomechanical differences may influence corneal response to applanation and provide a plausible explanation for the observed variability in the performance of GAT correction formulas across procedures. Consistent with this interpretation, the difference in DCT measurements before and after tPRK surgery is smaller (p > 0.05), and fewer correction formulas showed outstanding performance for tPRK compared with FS-LASIK or SMILE. It should be noted that the relatively smaller biomechanical alteration observed after tPRK may lead to reduced underestimation of GAT-measured IOP. Under these circumstances, correction formulas incorporating preoperative IOP (e.g., F1) may appear to perform better, partly because postoperative GAT values remain closer to baseline measurements rather than due to superior correction of surgery-induced biomechanical changes.

De Bernardo et al. compared postoperative GAT-IOP values (corrected by nine different formulas) with preoperative IOP values after myopic PRK and found that F13 (Rashad’s) (Rashad and Bahnassy, 2001) and F1 (Chihara’s) (Chihara et al., 2005) performed better when preoperative IOP was known, whereas F5 (Rosa’s) (Rosa et al., 1998) and F4 (Duch’s) (Duch et al., 2001) were superior when it was not. When we expanded the number of collected formulas and conducted a more comprehensive evaluation, we observed results that aligned partially with their findings. Specifically, F1 (Chihara’s) exhibited stronger applicability after tPRK, indicating that F1 maintains good stability even with a different study population.

Yang et al. compared 1-month postoperative IOP corrected by Pentacam’s five formulas (Ehlers, Shah, Dresden (F10), Orssengo Pye, and Kohlhaas (F7)) with their own preoperative IOP values using paired T-test for FS-LASIK and SMILE. (Yang et al., 2022). According to their study, the Shah formula appears to be the most suitable for SMILE, while the F10 (Dresden), Orssengo-Pye formula, and F7 (Kohlhaas) are better for FS-LASIK; notably, F7 was appropriate for FS-LASIK but not for SMILE. Since some of these formulas were not specifically designed for post-refractive surgery, we only included F10 (Dresden’s) and F7 (Kohlhaas’s) in our study. We evaluated the 14 formulas and assessed IOP at 3 months post-operation. Compared to 1 month after surgery, corneal edema had subsided and the cornea was more stable, which may result in a more stable 1OP. In our study, F7 (Kohlhaas’s) performed well after SMILE surgery and had relatively good correction results after FS-LASIK surgery, while F10’s advantage was not obvious.

Real IOP cannot be measured unless invasively measured, and it is uncertain whether the actual IOP changes after 3 months postoperatively. We assumed that ocular surface procedures in refractive surgery have minimal impact on real IOP, so we use preoperative IOP as the reference for corrected postoperative measurements. Some existing studies have similarly used preoperative IOP values as reference standards. (Duch et al., 2001; Rashad and Bahnassy, 2001; De Bernardo et al., 2016; Kohlhaas et al., 2006a). However, since the measurements of IOP before and after surgery were not taken on the same day, fluctuations in daily IOP could potentially affect the measurement results. Moreover, baseline differences in ablation depth and corneal curvature across surgical groups, reflecting intrinsic characteristics of different refractive procedures, may influence postoperative corneal biomechanics and thereby affect GAT measurements. Accordingly, the performance of correction formulas was assessed within each surgical group rather than through direct intergroup comparisons. Nevertheless, differences in baseline may partially account for the variability in formula performance among surgical techniques, which should be considered a limitation of the present study.

In conclusion, F3, F4, and F11 are more applicable in both FS-LASIK and SMILE, but do not show an advantage in tPRK. F1 and F2 are more appropriate in tPRK. Although F1 corrected measurement also improved in FS-LASIK and SMILE compared to uncorrected—yielding differences closer to DCT—its inclusion of preoperative IOP parameters, which was also our reference of comparison, may introduce evaluation bias. When preoperative IOP values are uncertain, it is recommended to use F2 for post-tPRK correction. Overall, our findings provide supportive methodological information for the selection of GAT correction formulas following different refractive surgical procedures and may be helpful during the screening stage for ocular hypertension after refractive surgery.

## Data Availability

The raw data supporting the conclusions of this article will be made available by the authors, without undue reservation.
